# A new twist on an old idea part 2: cyclosporine preserves normal mitochondrial but not cardiomyocyte function in mini‐swine with compensated heart failure

**DOI:** 10.14814/phy2.12050

**Published:** 2014-06-24

**Authors:** Jessica A. Hiemstra, Manuel Gutiérrez‐Aguilar, Kurt D. Marshall, Kyle S. McCommis, Pamela J. Zgoda, Noelany Cruz‐Rivera, Nathan T. Jenkins, Maike Krenz, Timothy L. Domeier, Christopher P. Baines, Craig A. Emter

**Affiliations:** 1 Department of Biomedical Science, University of Missouri‐ Columbia, 1600 E. RollinsW160 Veterinary Medicine, Columbia, Missouri; 2 Dalton Cardiovascular Research Center, University of Missouri‐ Columbia, 1600 E. RollinsW160 Veterinary Medicine, Columbia, Missouri; 3 Department of Medical Pharmacology and Physiology, University of Missouri‐ Columbia, 1600 E. RollinsW160 Veterinary Medicine, Columbia, Missouri; 4 Department of Kinesiology, University of Georgia, Athens, Georgia

**Keywords:** Calcium, cardiomyocyte, cyclosporine, HFpEF, mitochondria

## Abstract

We recently developed a clinically relevant mini‐swine model of heart failure with preserved ejection fraction (HFpEF), in which diastolic dysfunction was associated with increased mitochondrial permeability transition (MPT). Early diastolic function is ATP and Ca^2+^‐dependent, thus, we hypothesized chronic low doses of cyclosporine (CsA) would preserve mitochondrial function via inhibition of MPT and subsequently maintain normal cardiomyocyte Ca^2+^ handling and contractile characteristics. Left ventricular cardiomyocytes were isolated from aortic‐banded Yucatan mini‐swine divided into three groups; control nonbanded (CON), HFpEF nontreated (HF), and HFpEF treated with CsA (HF‐CsA). CsA mitigated the deterioration of mitochondrial function observed in HF animals, including functional uncoupling of Complex I‐dependent mitochondrial respiration and increased susceptibility to MPT. Attenuation of mitochondrial dysfunction in the HF‐CsA group was not associated with commensurate improvement in cardiomyocyte Ca^2+^ handling or contractility. Ca^2+^ transient amplitude was reduced and transient time to peak and recovery (tau) prolonged in HF and HF‐CsA groups compared to CON. Alterations in Ca^2+^ transient parameters observed in the HF and HF‐CsA groups were associated with decreased cardiomyocyte shortening and shortening rate. Cellular function was consistent with impaired in vivo systolic and diastolic whole heart function. A significant systemic hypertensive response to CsA was observed in HF‐CsA animals, and may have played a role in the accelerated the development of heart failure at both the whole heart and cellular levels. Given the significant detriment to cardiac function observed in response to CsA, our findings suggest chronic CsA treatment is not a viable therapeutic option for HFpEF.

## Abstract (precis)

In a recently developed a translational mini‐swine model of heart failure with preserved ejection fraction (HFpEF), we hypothesized inhibiting mitochondrial permeability transition using cyclosporine (CsA) would improve cardiomyocyte function and calcium handling by supporting mitochondrial function. The purpose of this study was to examine the impact of inhibiting cyclophilin D on mitochondrial function and subsequent cardiomyocyte calcium handling using a reduced, nonimmunosuppressive dose of CsA chronically. We found improved mitochondrial function following chronic CsA treatment was not associated with a parallel improvement in cardiomyocyte calcium handling and contractile function, and demonstrate for the first time impaired cardiomyocyte calcium handling and contractile function are present early in the disease process in our HFpEF model.

## Introduction

Approximately one half of heart failure (HF) patients demonstrate normal left ventricular (LV) function at rest, a condition clinically recognized as heart failure with preserved ejection fraction (HFpEF). Conventional treatments commonly used for heart failure with reduced ejection fraction (HFrEF) patients have proven ineffective for HFpEF, highlighting the critical need for development of novel treatment options for this HF subpopulation (Maeder and Kaye 2009; Paulus and van Ballegoij 2010; Borlaug and Paulus 2011; Borlaug and Redfield 2011; Burkhoff 2012; Zile et al. 2013). Mitochondrial dysfunction, impaired myocardial energetics, and cell death are critically connected during developing heart failure, and the mitochondrial permeability transition (MPT) pore is thought to play a key role in this pathological process (Zhang 2002; Ventura‐Clapier et al. 2004; Foo et al. 2005; Dorn 2009; Javadov et al. 2009). Indeed, our group has previously shown mitochondrial dysfunction, specifically increased susceptibility to MPT, is present in the early stages of a clinically relevant miniature swine model of HFpEF (induced by aortic banding and subsequent pressure overload) and may play a causal role in the development of terminal decompensated HF (Emter and Baines 2010).

While impaired cardiomyocyte Ca^2+^ handling and contractile function is a consistent finding in HFrEF (Louch et al. 2012), changes in Ca^2+^ handling and contractile function during HFpEF‐related disease is more complex. In rodent and small animal models of hypertrophy and heart failure induced by chronic increases in afterload (e.g., aortic banding or hypertension), Ca^2+^ transient amplitude and contractility has been shown to be both increased (Brooksby et al. 1993; Shorofsky et al. 1999) and decreased (Siri et al. 1991; Bailey and Houser 1992; Gomez et al. 1997; Diaz et al. 2004) depending on etiology of disease, degree of cardiac compensation, and stage of HF progression (Kapur et al. 2010). In the healthy heart, intracellular Ca^2+^ and cellular energetic state are critically linked allowing the cardiomyocyte to increase cellular ATP production in response to increased Ca^2+^ cycling that arises as a result of increased heart rate and/or adrenergic stimulation. Given the well‐established calcium sensitivity of the MPT pore, impaired mitochondrial function and calcium mishandling in heart failure could play a critical role in our recent report of diastolic dysfunction in the same mini‐swine HFpEF model (Marshall et al. 2013). In total, our previous results suggest the presence of a pathological positive feedback loop early in the pathogenesis of HFpEF in which impaired mitochondrial function may exacerbate cardiomyocyte calcium mishandling, subsequently enhancing susceptibility to MPT and potentiating the development of LV dysfunction in HF.

Although the molecular components of the MPT pore are still a matter of debate (Bernardi 2013), cyclophilin D (CypD) is widely accepted as a key component the pore. Therefore, the purpose of this study was to examine the impact of inhibiting CypD on mitochondrial function and subsequent cardiomyocyte calcium handling using cyclosporine A (CsA). Inhibiting calcineurin and related cardiac remodeling in heart failure via the use of immunosuppressive doses of CsA has been comprehensively examined (Frey and Olson 2003; Wilkins et al. 2004). Indeed, the use of CsA analogues has progressed to the phase III CIRCUS clinical trial that has demonstrated an acute dose of CsA reduces reperfusion injury after acute myocardial infarction (Piot et al. 2008). In contrast, we used a novel approach compared to previous work by employing a reduced, nonimmunosuppressive dose of CsA chronically with the intent to inhibit CypD without subsequent interference of calcineurin signaling or associated cardiac remodeling (Okumi et al. 2008; Piot et al. 2008; Rigol et al. 2008; Marechal et al. 2011). Our laboratory recently reported the effects of CsA on cardiac remodeling and function in vivo (Hiemstra et al. 2013), and this study is specifically focused on the cardiomyocyte molecular response in these same animals. To examine these ideas, we used our model of HFpEF that displays key pathological features associated with the disease including diastolic dysfunction, depressed contractile reserve, fibrosis, hypertrophy, and increased natriuretic peptide expression (Marshall et al. 2013). We hypothesized that (1) previous observations of mitochondrial dysfunction in our model would be associated with impaired cardiomyocyte Ca^2+^ handling and; (2) our CsA dosing scheme would preserve normal mitochondrial function via inhibition of MPT and as a result, maintain normal cardiomyocyte Ca^2+^ handling and contractile characteristics.

## Methods

### Aortic banding and cyclosporine treatment

The animals in this study are the same animals utilized in recently published work from our laboratory in *Physiological Reports* (Hiemstra et al. 2013), and the following methods are presented *verbatim* for reference. “Briefly, before aortic banding intact male Yucatan miniature swine (27–30 kg; 8 months old) was matched for body mass and cardiac function then assigned into three groups: non–sham sedentary control (CON; *n* = 5), banded HF sedentary (HF; *n* = 5), and banded HF CsA treated (HF‐CsA; *n* = 5). Heart failure was induced by aortic banding for a period of 20 weeks using methods previously published by our laboratory (Marshall et al. 2013). All animals were sacrificed at this time point for experiments. A systolic trans‐stenotic gradient of approximately 70 mmHg (73 ± 2, 74 ± 1, for HF and HF‐CsA, respectively, P = NS) was achieved while maintaining a distal peripheral vascular MAP of approximately 90 mmHg (93 ± 1, 90 ± 1, for HF and HF‐CsA, respectively, P = NS) under anesthesia using phenylephrine (I.V. 1–3 μg/kg/min) at a heart rate of 100 beats/min (100 ± 5, 107 ± 2, for HF and HF‐CsA, respectively, P = NS). Following the development of left ventricular (LV) hypertrophy, treatment with CsA (2.0 mg/kg/day, oral) or placebo began 6 weeks postaortic banding and continued daily for 14 weeks. Animals were fed a standard diet averaging 15–20 g/kg once daily, and water was provided ad libitum. Dissection of vital tissues occurred at the time of death. All animal protocols were in accordance with the ‘Principles for the Utilization and Care of Vertebrate Animals Used in Testing Research and Training’ and approved by the University of Missouri Animal Care and Use Committee.”

### Calcineurin activity

Calcineurin activity was measured using the Calcineurin Cellular Activity Assay Kit, colorimetric (EMD Millipore, Billerica, MA) according to manufacturer's instructions. Briefly, cardiomyocyte lysates were incubated with RII phosphopeptide (Asp‐Leu‐Asp‐Val‐Pro‐Ile‐Pro‐Gly‐Arg‐Phe‐Asp_Arg‐Arg‐Val‐pSer‐Val‐Ala‐Ala‐Glu) in reaction buffer either in the presence or in the absence of 20 mmol/L EGTA. Release of inorganic phosphate was determined colorimetrically. After background correction, calcineurin activity was calculated as follows: calcineurin‐specific activity = activity without EGTA – activity in the presence of EGTA.

### Serum cytokine concentrations

Serum from fasting animals were assayed in duplicate for concentrations of Interleukin‐2 (IL‐2), IL‐6, IL‐10, IL‐12, and IL‐18 using a porcine specific multiplex cytokine/chemokine assay (Millipore Milliplex, cat. PCYTMAG‐23K; Billerica, MA) on a MAGPIX instrument (Luminex Technologies; Luminex, Austin, TX) according to the manufacturer's instructions.

### Tissue fractionation

Heart subcellular fractions were prepared from pig left ventricle (LV) by differential centrifugation in sucrose‐based medium, as previously described (McCommis et al. 2011). Briefly, 3 g of left‐ventricular tissue was homogenized using a Dounce in homogenization buffer (250 mmol/L sucrose, 10 mmol/L Tris pH 7.4, and 1 mmol/L EDTA). The homogenate was centrifuged at 1000 g for 5 min to pellet the nuclei and unbroken cells/debris and further filtered to measure calcineurin activity. The supernatant was centrifuged at 10,000 g for 10 min to pellet the mitochondria. The cytosolic fraction was then prepared by centrifuging the postmitochondrial supernatant at 20,000 g for 30 min. The mitochondrial pellet was then washed twice in EDTA‐free homogenization buffer and resuspended in lysis buffer (150 mmol/L NaCl, 10 mmol/L Tris pH 7.4, 1 mmol/L EDTA, and 1% Triton X‐100). Protein concentration was determined by Bradford assay (Bio‐rad, Hercules, CA).

### Mitochondrial swelling and calcium retention

Mitochondria were resuspended in isosmotic swelling buffer (120 mmol/L KCl, 10 mmol/L Tris at pH 7.4 and 5 mmol/L KH_2_PO_4_) at 0.20 mg/mL in a final volume of 0.8 mL (Baines et al. 2005). MPT pore opening was measured spectrophotometrically as a decrease in light scattering at 520 nm and was induced by the addition of 25 μmol/L CaCl_2_ under de‐energized conditions. Swelling was monitored for 10 min and results are shown as representative traces following quantification of area above the curve analysis (in absorbance units*min). For calcium retention capacity experiments, 0.10 mg/mL of mitochondrial protein was resuspended in calcium retention buffer (120 mmol/L KCl, 10 mmol/L Tris at pH 7.4, 1 mmol/L KH_2_PO_4_ and 0.02 mmol/L EDTA) plus 1 μmol/L Ca^2+^‐green 5N (Molecular Probes/Life Technologies, Grand Island, NY). Changes in fluorescence at 530 nm were recorded in a 1 mL cuvette using a fluorimeter with constant stirring of the sample. Sequential pulses of 5 μmol/L Ca^2+^ were added each minute until a massive fluorescence increase, indicative of MPT pore‐induced Ca^2+^ release, was detected (Gutierrez‐Aguilar et al. 2014).

### Respiratory chain activity

Oxygen consumption was measured polarographically at 25°C using a Clark‐type electrode in the medium used for swelling measurements supplemented with 1 mmol/L MgCl_2_ and either 5 mmol/L glutamate and 5 mmol/L malate to measure mitochondrial Complex I‐dependent respiration or 10 mmol/L succinate to measure mitochondrial Complex II‐dependent respiration. Following the addition of 0.125 mg/mL of mitochondrial protein to the oxygen consumption cuvette, State 2 (resting state) was recorded. State 3 was initiated by adding 0.2 mmol/L ADP to the reaction mixture 200 sec after recording State 2 respiration. Once the respiratory rates were determined, the State3/State2 respiratory control (R.C.) ratio was obtained.

### Western blotting

Tissue was homogenized as described previously in buffer containing 150 mmol/L NaCl, 10 mmol/L Tris (pH 7.4), 1 mmol/L EDTA, and 1% Triton‐X100 (McCommis et al. 2011). Proteins were resolved by SDS‐PAGE using 10% acrylamide, transferred onto PVDF membranes, and immunoblotted using the following commercially available antibodies (all at 1:1000): anti‐CypD and LDH from Abcam (Cambridge, MA); anti‐PiC from YenZym (San Franciso, CA); anti‐ANT and Actin from Santa Cruz (Dallas, TX); anti‐VDAC from BD Biosciences (San Jose, CA); Oxphos antibody cocktail from Abcam; anti GAPDH from Sigma (St. Louis, MO); GAPDH and NFAT4 from Cell Signaling. Membranes were then incubated with the appropriate alkaline phosphatase‐linked secondary antibody from Cell Signaling and visualized by enhanced chemifluorescence (Amersham, Pittsburgh, PA). To determine nuclear translocation of NFAT4, nuclear and cytosolic subfractions were obtained by differential centrifugation. The homogenate was centrifuged at 1000 g for 5 min to pellet the nuclei and unbroken cells/debris. The pellet was resuspended in 5 mL homogenization buffer and filtered with a 40 μm nylon mesh filter (Fisher Scientific, Waltham, MA). Two mL of the filtered fraction was equally divided into two Eppendorf tubes and washed twice. Pellets were resuspended in 400 μL of homogenization buffer, and 20 μL 20% TX100 was added to the samples (prior to sonication and centrifugation) to pellet insoluble material. The soluble fractions were further considered for nuclear translocation of NFAT4 measurements.

### Cardiomyocyte cytosolic Ca^2+^ ([Ca^2+^]_i_) measurements

Left‐ventricular cardiac myocytes were isolated from a left‐ventricular wedge preparation as previously described (McDonald et al. 2012). Isolated myocytes plated on laminin‐coated 24 × 50 mm glass coverslips were loaded with 5 μmol/L fluo‐4/AM (Molecular Probes) for 10 min followed by a 30–50 min wash at room temperature. Coverslips were superfused with physiological saline solution containing (in mmol/L): 135 NaCl, 5 KCl, 2 CaCl_2_, 1 MgCl_2_, 10 D‐glucose, 10 Hepes, 1 mmol/L NaHCO_3_, pH 7.4 with NaOH. Bath temperature was controlled at 34–36°C via an inline heater (SHM828; Warner Instruments, Hamden, CT) and temperature‐controlled heating element (PH‐1; Warner Instruments). Epifluorescence microscopy was performed using an inverted fluorescence microscope (IX71; Olympus America, Center Valley, PA) with a 40× oil‐immersion objective (UApo/340: 1.35 NA, Olympus), with excitation at 460–480 nm and emission collected at 500–550 nm. An individual cardiomyocyte was positioned within a recording window (~275 × 75 μm) and fluorescence emission and sarcomere length (L_s_) were simultaneously sampled using an IonOptix Calcium and Contractility Recording System (IonOptix, Milton, MA). Action potentials (0.25, 0.5, and 1 Hz) were induced using electrical field stimulation (S48; Grass Instruments, Warwick, RI) with platinum electrodes placed at the edges of the bath. Action potential‐induced Ca^2+^ transients and L_s_ shortening were analyzed offline using Ionwizard 6.3 software (IonOptix) with the following parameters assessed: (1) Ca^2+^ transient amplitude (*ΔF/F*_*0*_, where F_0_ is the fluorescence prior to the Ca^2+^ transient and ΔF = F_peak_‐F_0_); (2) Ca^2+^ transient time to peak; (3) Ca^2+^ transient recovery (*tau*, determined using an exponential fit to the fluorescence signal decay between 50% of peak and the baseline); (4) Percent shortening ((1‐(L/L_0_)) × 100), where L_0_ is the length prior to shortening and L the minimum length after shortening; and (5) maximum rate of shortening ΔL/Δt (μm/sec). Values of 3–6 consecutive steady‐state transients were obtained and averaged to obtain a single average value for each parameter per cell per experimental trial. All fluorescence values were background subtracted prior to analysis.

### Histology and immunohistochemistry

Cross sections of LV were formalin fixed, embedded in paraffin, and stained for assessment of fibrosis and collagen. Briefly, total fibrosis was visualized from 4‐μm‐thick sections of LV using Masson's trichrome stain, and total collagen was visualized using Picrosirus red staining with methods previously established (Emter and Baines 2010; Marshall et al. 2013). Fibrosis and collagen were quantified from four separate fields/animal using Image‐Pro Plus analysis software (MediaCybernetics, version 6.2, Bethesda, MD) and expressed as the percent area stained. To optimize visual presentation, color balance of acquired images was adjusted according to RGB pixel intensity histograms using ImageJ software (NIH). Images from the three respective groups were set at identical RGB pixel values.

### Statistical analysis

All data analysis was performed using SPSS version 19.0 (IBM Corporation, Armonk, NY) or SigmaStat version 3.5 (Systat Software Inc., San Jose, CA). Power analyses were conducted to determine the number of animals needed as recommended by Kim and Seo (2013) using the Sealed Envelope Power Calculator (http://www.sealedenvelope.com/power/continuous-superiority/). For input, previously obtained mitochondrial swelling data from five CON and five HF pigs were used to ensure the study was sufficiently powered to detect changes in MTP opening. Significance level was set at 5%, power at 90%, mean outcome in control group 0.08, mean outcome in experimental group 0.21, and standard deviation of outcome 0.05. The power analysis showed that *n* = 4 per group would be sufficient to detect differences, therefore, group size was set at five animals. Group comparisons were made using either one‐way or repeated measures ANOVA. Group differences revealed by ANOVA were found using Student Newman–Keuls post hoc analysis. Within group comparisons were made using paired samples *t*‐test. All data are presented as means ± SE, and significance is reported at *P* < 0.10 and *P* < 0.05 levels (Williams et al. 1997; Curran‐Everett and Benos 2004; Emter et al. 2005, 2011; Emter and Baines 2010; Hiemstra et al. 2013; Marshall et al. 2013).

## Results

### LV remodeling, function, and hemodynamics

The animals in this study are the same animals utilized in recently published work from our laboratory in *Physiological Reports* (Hiemstra et al. 2013), where we provide a thorough in vivo examination of myocardial function using a comprehensive combination of techniques including computed tomography (CT), 2‐D speckle tracking, and invasive hemodynamics. Here, we provide a brief summary of these results, which indicated our CsA dosing scheme accelerated the development of early evidence for heart failure including dilatory LV remodeling and impaired systolic and diastolic mechanics. Concentric LV hypertrophy, evident by significantly increased LV diastolic wall thickness in the absence of alterations to LV end diastolic dimension, was present 1–2 months postsurgery in all aortic‐banded groups prior to the onset of CsA treatment. Following CsA treatment, CT assessment of LV morphology indicated LV mass, end systolic volume (LV ESV), end diastolic volume (LV EDV), and left atrial (LA) volume were significantly increased in the HF‐CsA group. Normalization of LV mass showed an increase in only the HF group (assessed by both CT and postmortem) indicating although LV remodeling occurred in both HF groups, hypertrophy was concentric in HF animals as opposed to eccentric (i.e., dilated) in the HF‐CsA group. Resting hemodynamic and LV functional data following CsA treatment demonstrated a general depression of systolic function in HF‐CsA animals compared to CON and HF groups. In HF‐CsA animals, LV ejection fraction (EF%) was reduced compared to CON and HF groups and observed in parallel with increased LV ESV. Left ventricular end systolic pressure (65 ± 7, 81 ± 6, and 109 ± 9 mmHg for CON, HF, and HF‐CsA, respectively), LV end diastolic pressure (11 ± 1, 11 ± 1, and 15 ± 1 mmHg for CON, HF, and HF‐CsA, respectively), and peripheral mean arterial pressure (distal to the aortic band; 53 ± 11, 59 ± 4, and 86 ± 8 mmHg for CON, HF, and HF‐CsA, respectively) was significantly increased in HF‐CsA compared to HF and CON groups, suggesting a hypertensive reaction to CsA treatment. 2‐D speckle tracking detected LV mechanics associated with systolic and diastolic dysfunction including decreased systolic rotation rate, decreased systolic longitudinal/radial/circumferential strain, decreased early diastolic untwisting and longitudinal strain rate, and increased late diastolic radial/circumferential mitral strain rate.

### Calcineurin activity and inflammation

**Figure 1.**
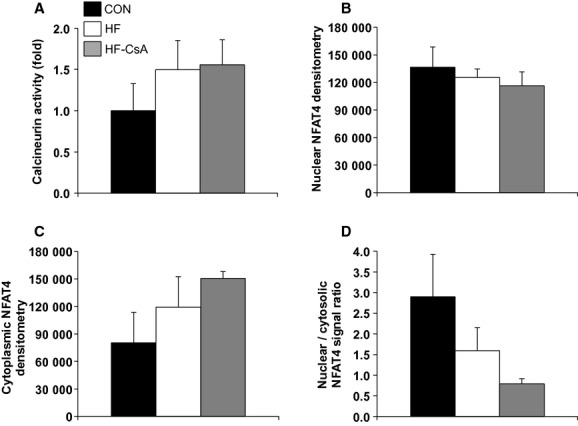
Calcineurin activity and NFAT protein expression (*N* = 5 for each group). (A) Treatment with CsA does not suppress calcineurin activity. (B–D) CsA did not reduce NFAT4 protein expression in the nucleus (B), cytoplasm (C), or as a ratio of these cellular domains (D).

**Figure 2.**
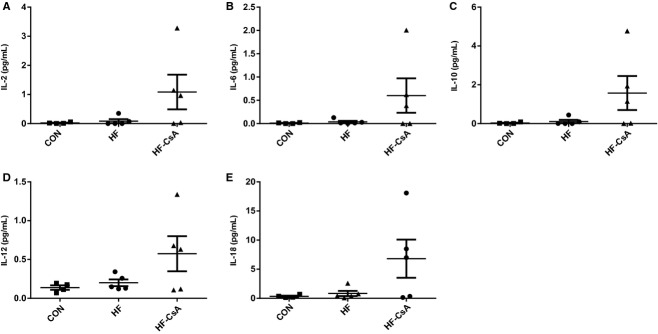
Systemic inflammation (*N* = 5 for each group). (A–E) CsA did not cause immunosuppression as reflected in the plasma expression of several inflammatory interleukin isoforms (IL‐2, IL‐6, IL‐10, IL‐12, and IL‐18).

The effects of our CsA dosing scheme on calcineurin activity and the immune response are presented in Figures 1 and 2, respectively. Neither calcineurin activity (Fig. 1A) nor nuclear (Fig. 1B) / cytoplasmic (Fig. 1C) protein expression of the calcineurin target, NFAT4, were altered in HF‐CsA animals compared to the CON and HF groups. There were also no significant reductions in circulating plasma cytokines (IL‐2, IL‐6, IL‐10, IL‐12, and IL‐18; Fig. 2A–E) in the HF‐CsA group compared to CON or HF. In total, these results indicate we were successful in avoiding immunosuppression and inhibition of calcineurin following treatment with CsA.

### Mitochondrial function

**Figure 3.**
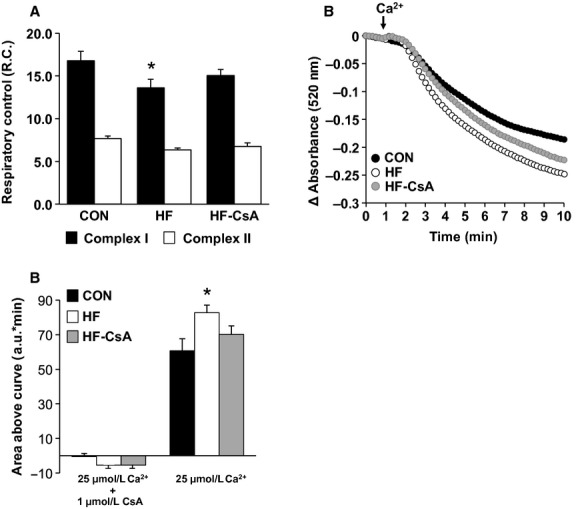
Mitochondrial respiratory control and susceptibility to permeability transition (*N* = 5 for each group). (A) CsA treatment attenuated a heart failure‐induced decrease in mitochondrial complex I‐dependent respiratory coupling (black bars). (B–C) CsA reduced an increased susceptibility to Ca^2+^‐induced mitochondrial swelling, an index of MPT, observed in the HF group. Quantification of changes in absorbance in the presence of 25 μmol/L Ca^2+^ with or without CsA are presented in 3C as area above the curve of the traces in *3B*. (*P < 0.05 vs. CON).

Oxygen consumption (using respiratory Complex I and II substrates) and permeability transition experiments in isolated LV mitochondria are presented in Figure 3. In the presence of respiratory Complex I substrates, R.C. was significantly lower in mitochondria from the HF group compared to CON (Fig. 3A – black bars). This effect was attenuated by CsA treatment. In the presence of succinate as an electron donor, mitochondria from the CON, HF and HF‐CsA groups presented statistically similar R.C. (Fig. 3A – white bars).

**Figure 4.**
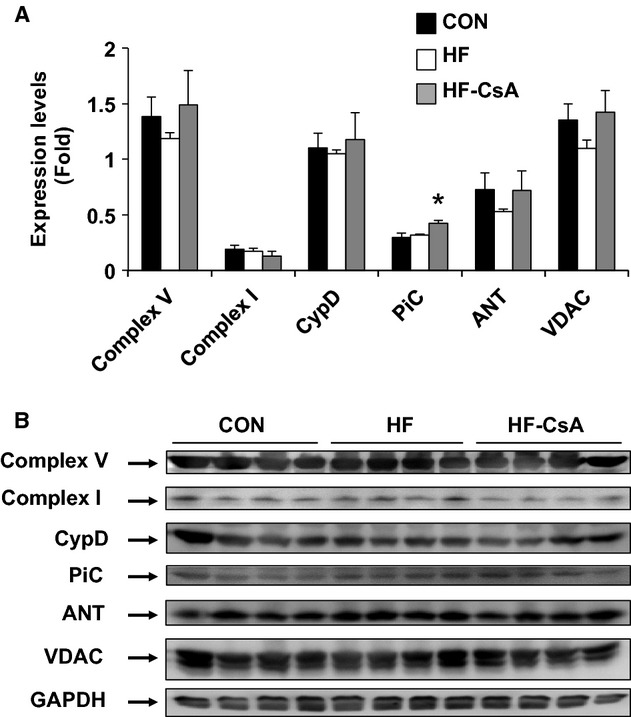
Western blot analysis of putative components thought to comprise the MPT pore in cardiac mitochondria (*N* = 5 for each group). (A) Protein expression of PiC (the mitochondrial Phosphate Carrier) was increased in LV from the HF‐CsA group. No group differences in ATP synthase (Complex V), cyclophilin‐D (CypD), Complex I (NADH:ubiquinone oxidoreductase), voltage‐dependent anion channel (VDAC), or adenine nucleotide translocase (ANT) expression were observed. Protein levels were normalized to glyceraldehyde 3‐phosphate dehydrogenase (GAPDH) to control for intragroup variability. (B) Representative western blots for each protein target. (*P < 0.05 vs. CON & HF).

To gain insight into the nature of the heart failure‐mediated mitochondrial uncoupling and the protective effects of CsA, we determined the sensitivity of isolated mitochondria to Ca^2+^‐induced MPT pore‐dependent mitochondrial swelling (Fig. 3B). Mitochondria from the HF group swelled to a greater extent compared to CON animals in the presence of 25 μmol/L Ca^2+^ and was quantitatively confirmed by area‐above‐the‐curve analyses (Fig. 3C). Chronic treatment with CsA attenuated the increased susceptibility to MPT associated with developing heart failure. We further examined whether observed changes in the MPT response were derived from variations in the expression of proteins thought to comprise the MPT pore including mitochondrial Complex V, Complex I, CypD, mitochondrial Phosphate Carrier (PiC), and Adenine Nucleotide Translocase (ANT) (Fig. 4A). The attenuation of mitochondrial dysfunction in the HF‐CsA group was associated with a significant increase in PiC protein expression compared to CON and HF animals (Fig. 4A). Western blots performed on LV homogenates were normalized to GAPDH expression to control for intragroup variability.

### Cardiomyocyte calcium handling and contractility

**Figure 5.**
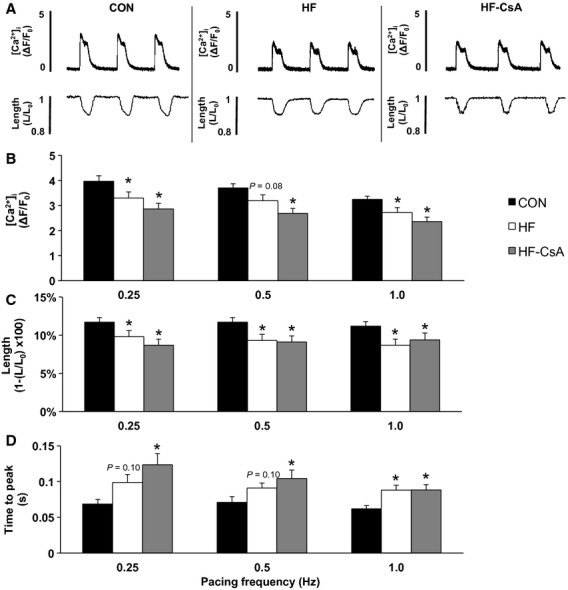
Cardiomyocyte frequency dependence of calcium transients and cell shortening (CON: *n* = 3 animals, 20 cells & HF: *n* = 5 animals, 20 cells, for all measures; HF‐CsA: *n* = 5 animals, 25 cells for all calcium measures & 15 cells for all shortening measures). (A) Representative cardiomyocyte calcium and shortening transients from CON (left), HF (center), and HF‐CsA (right) animals. (B) Calcium ([Ca^2+^]_I_) transient amplitude (ΔF/ F_0_) and (C) percent shortening (1‐L / L_0_) were decreased in both HF and HF‐CsA groups compared to CON at all pacing frequencies. (D) Time to peak of the calcium transient was increased in HF and HF‐CsA animals compared to the CON group. (*P <**0.05 vs. CON).

**Figure 6.**
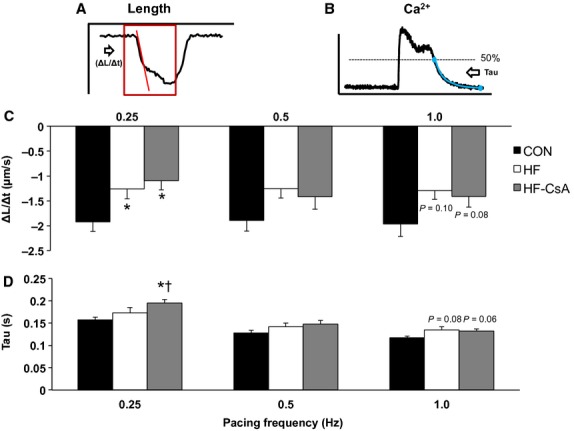
Cardiomyocyte shortening rate and cystolic calcium reuptake (CON: *n* = 3 animals, 20 cells & HF: *n* = 5 animals, 20 cells, for all measures; HF‐CsA: *n* = 5 animals, 25 cells for all calcium measures & 15 cells for all shortening measures). (A–B) Representative traces depicting methods used to measure rate of shortening and calcium reuptake (Tau), respectively. (C) Cardiomyocyte shortening rate (ΔL/Δt) was slower (less negative) in HF and HF‐CsA animals at pacing frequencies of 0.25 and 1.0 Hz. (D) Cytosolic calcium reuptake (Tau) was increased in HF‐CSA animals at 0.25 Hz and both HF groups at 1.0 Hz compared to CON. (*P <**0.05 vs. CON; ^†^*P *<**0.05 vs. HF).

Calcium and cell shortening transients were analyzed in isolated LV cardiomyocytes (CON: *n* = 3 animals, 20 cells; HF: *n* = 5 animals, 20 cells; HF‐CsA: *n* = 5 animals, 15–25 cells) paced at increasing frequencies (0.25, 0.50, and 1.0 Hz). Frequency dependence of calcium transient amplitude, time to peak of the calcium transient, calcium transient decay (tau), percent shortening, and rate of shortening (ΔL/Δt) were measured. Representative calcium and shortening transients from each group are presented in Figure 5A. Calcium transient amplitude (*ΔF/F*_*0*_) was significantly decreased in HF and HF‐CsA animals (Fig. 5B) and observed in parallel with a significant increase in time to the peak of the calcium transient (Fig. 5D) in both HF groups regardless of frequency. Cell contraction, measured by percent shortening (Fig. 5C) and rate of shortening (ΔL/Δt, Fig. 6C), was also decreased in HF and HF‐CsA animals at almost all pacing frequencies. A documented feature of the porcine cardiomyocyte is the spike and dome morphology of the calcium transient, previously described as a characteristic of cardiac M cells and caused by the reopening of L‐type calcium channels (Stankovicova et al. 2000). As a result, rate of intracellular calcium removal (tau) was measured from 50% recovery to baseline (Fig. 6B) allowing for an exponential fit to this phase of the transient. Tau was significantly increased at 0.25 Hz in the HF‐CsA group and at 1.0 Hz in both HF groups compared to CON (Fig. 6D). In summary, CsA treatment did not rescue the impairment in cardiomyocyte calcium handling or contraction observed in HF animals.

### LV fibrosis

**Figure 7.**
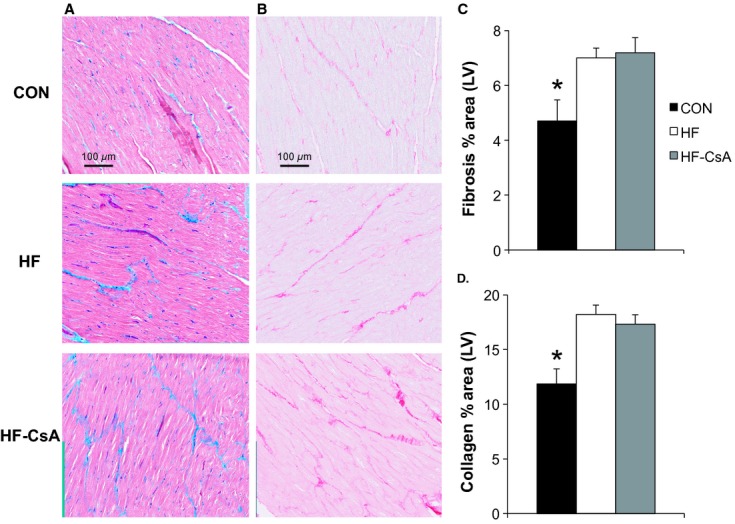
LV fibrosis and collagen deposition (*N* = 5 for each group). (A–B) representative histological sections of trichrome and Picrosirius red‐stained LV, showing increased fibrosis in HF and HF‐CsA animals. Magnification: ×40. (C–D) CsA treatment did not prevent an increase in total fibrosis and collagen induced by heart failure as indicated by assessment of percent area stained. (*P <**0.05 vs. HF & HF‐CsA).

Representative histological sections of the LV from CON, HF, and HF‐CsA animals are shown in Figures 7A and B. Trichrome and Picrosirus red staining (expressed as the percent area of LV stained) indicated general fibrosis and total collagen were significantly elevated in HF animals compared to CON (Figs 7C and D, respectively). CsA treatment did not prevent heart failure‐induced increases in LV fibrosis.

## Discussion

In this study, we examined the effects of a novel CsA dosing scheme on cardiomyoctye mitochondrial and contractile function, calcium handling, and ECM remodeling in a miniature swine model of HFpEF. Our results indicate (1) impaired cardiomyocyte calcium handling and contractile function are present early in the HF disease progression in our model; (2) chronic CsA treatment attenuated mitochondrial dysfunction, specifically susceptibility to MPT and decreased Complex I‐dependent respiration, during developing heart failure; (3) improved mitochondrial function was not associated with a parallel improvement in cardiomyocyte calcium handling or contractile function.

HFpEF comprises approximately 50% of all HF cases (Hunt et al. 2005; Maeder and Kaye 2009; Borlaug and Paulus 2011). Interestingly, conventional therapies used in HFrEF patients have proven ineffective for HFpEF, illustrating a critical need for novel therapeutic treatment options for this HF subgroup (Maeder and Kaye 2009; Paulus and van Ballegoij 2010; Borlaug and Paulus 2011; Borlaug and Redfield 2011; Burkhoff 2012). In contrast to previous work examining immunosuppressive doses of CsA as an inhibitor of calcineurin and associated cardiac remodeling during the development of HF, this study was specifically designed to avoid calcineurin inhibition and/or subsequent immunosuppression. The CsA dose used in this study (2 mg/kg/day) was based on the effective human dose used to treat ischemia / reperfusion injury and myocardial infarction in a preliminary clinical study (Piot et al. 2008) (which has now moved into the phase III CIRCUS clinical trial) and 5–10 times lower than the dose need to induce immunosuppression in pigs (10–20 mg/kg/day) (Okumi et al. 2008; Rigol et al. 2008). However, an important difference between our study and the CIRCUS trial is the use of CsA chronically as opposed to a single dose, respectively. Extensive work has demonstrated CypD is a key regulator of the MPT pore (Baines et al. 2005), thus, the CsA dosing scheme employed in this study was designed to inhibit mitochondrial CypD and subsequent MPT. We hypothesized that in the presence of sustained pressure overload inhibition of MPT would preserve normal mitochondrial function and as a result, maintain normal cardiomyocyte Ca^2+^ handling and contractile characteristics.

Indeed, a key finding of our study was the preservation of normal mitochondrial function following chronic treatment with CsA, consistent with our original hypothesis. Our finding of an enhanced susceptibility to MPT in HF animals in this study confirms previous results from our group (Emter and Baines 2010), and we expand on these findings in this study showing increased MPT is associated with decreased mitochondrial oxidative phosphorylation efficiency in the presence of Complex I substrates. These results correspond with recent work demonstrating a critical regulatory link between Complex I and the MPT pore (Li et al. 1817). The uncoupled mitochondrial phenotype observed in the HF group was attenuated in HF‐CsA animals, implying our CsA dosing scheme was effective in inhibiting CypD and subsequent MPT. The protective effects of CsA on mitochondrial respiration may rely on an enhanced ability to sequester higher levels of Ca^2+^ without triggering MPT and a commensurate activation of Ca^2+^‐dependent mitochondrial dehydrogenases of the Krebs cycle (Elrod et al. 2010). Furthermore, increases in mitochondrial matrix Ca^2+^ may also provide beneficial effects by replenishing the pool of reduced NADPH, thus, increasing mitochondrial antioxidant capacity (Kohlhaas et al. 2010). Given the enhanced sensitivity to Ca^2+^‐induced MPT associated with the development of heart failure in our model and the observed level of calcium mismanagement in all heart failure groups, it is reasonable to speculate that inhibition of CypD via the use of CsA in this study was an essential factor in attenuating mitochondrial dysfunction in HF‐CsA animals.

In addition to preserving normal mitochondrial function, our data also indicate we effectively accomplished our goals of not inhibiting calcineurin or suppressing the immune system. Neither calcineurin activity (Fig. 1A) nor nuclear NFAT protein expression (Fig. 1B), a downstream indicator of calcineurin signaling (Molkentin et al. 1998), was significantly altered in the HF‐CsA group compared to CON animals. Upregulation of the calcineurin‐NFAT signaling pathway has a well‐established role in the development of concentric hypertrophy in the diseased heart (Frey and Olson 2003; Semeniuk et al. 2003; Diedrichs et al. 2007) and in end stage human heart failure (Lim and Molkentin 1999; Haq et al. 2001; Diedrichs et al. 2004). Considered in this context, it is difficult to assess the general influence of calcineurin on pathological remodeling in our model. However, our results do suggest that an increase in calcineurin activity and/or related signaling is not crucial at all times to the development or maintenance of the compensated heart failure state. The expression of several circulating pro‐inflammatory cytokines was also not suppressed following CsA treatment (Fig. 2A–E). Interestingly, plasma cytokine levels actually trended higher in the HF‐CSA group. Although we did not explore this finding further given the direction of change was opposite of what we would have expected had our CsA dosing scheme caused suppression of the immune system, it is interesting that some of the interleukins we measured were markedly elevated in three of the five CsA‐treated treated pigs (Fig. 2). This paradoxical increase in systemic inflammation is one finding in this study (amongst others) suggesting the translation of chronic low CsA dosing as a novel treatment for human HFpEF would be unlikely.

Perhaps the most interesting finding of this study was our CsA dosing scheme accelerated the development of early evidence for heart failure, including dilatory LV remodeling and impaired systolic and diastolic mechanics, despite evidence of improved mitochondrial function. Our previously reported data (Hiemstra et al. 2013) (obtained in the same animals used in this study) showed dilatory remodeling of the LV was associated with altered LV mechanics related to reduced systolic emptying, impaired early diastolic filling and enhanced atrial systole that extended beyond the impairment observed in the HF group. The exacerbated deterioration of whole heart function following our CsA intervention was quite unexpected, particularly given the deterioration of mitochondrial function was attenuated in the HF‐CsA group. As a result, we considered whether this deleterious outcome following CsA treatment was possibly the result of an unanticipated effect on cardiomyocyte calcium handling, contractile function, or ECM remodeling.

Again to our surprise, cardiomyocyte calcium handling and contractile function were impaired to a similar degree in both HF groups suggesting these mechanisms were not responsible for the accelerated development of heart failure following CsA treatment. These findings refuted our initial hypothesis, suggesting preservation of normal mitochondrial function may be insufficient to prevent a hallmark feature of heart failure, impaired cardiomyocyte calcium handling. A common characteristic of HFpEF observed in our model is a reduction in cardiac reserve (Tan et al. 2009; Norman et al. 2011; Marshall et al. 2013). In this study, cardiomyocytes were paced at increasing frequencies (0.25, 0.5, and 1 Hz) as a method to indirectly assess cardiac reserve in vitro. Our results revealed decreased calcium transients and contractility were observed in parallel to a similar degree in both the HF and HF‐CsA groups (Figs 5 and 6). Cardiomyocyte function correlated with depressed systolic LV mechanics previously observed in these same animals (Hiemstra et al. 2013), demonstrating cohesiveness between in vivo whole heart mechanical and cellular contractile function. Interestingly, evidence of depressed systolic mechanics was present in all HF groups. While these findings appear counterintuitive, the coexistence of diagnostic indices of both normal and reduced systolic function is common and difficult to reconcile in HFpEF (Tan et al. 2009; Norman et al. 2011; Hiemstra et al. 2013; Marshall et al. 2013) and results from this study contribute to this interesting and often observed paradox.

Diastolic dysfunction is another key feature of HFpEF critically linked to the management of intracellular calcium, and we observed a significant pacing‐induced reduction in the rate of cytosolic calcium removal in both HF and HF‐CsA animals (Fig. 6D). The reduced rate of cytosolic calcium removal observed in both HF groups, specifically measured as an increase in Tau, may be due to a decrease in sarcoplasmic/endoplasmic reticulum Ca^2+^ ATPase (SERCA) activity. Indeed, substantial evidence exists that decreased expression and/or function of SERCA or accessory proteins (e.g., phospholamban) impairs cytosolic Ca^2+^ removal in HF (Kho et al. 2012). Decreased SERCA function may also lead to depletion of the SR Ca^2+^ store, a decrease in Ca^2+^ transient amplitude, and a decrease in subsequent contractile function. We have previously shown contractile reserve is diminished in response to β‐adrenergic stimulation in this HFpEF model (Marshall et al. 2013), suggesting the normal downstream effects of cAMP‐PKA signaling on phospholamban and subsequent SERCA activity could underlie reductions in diastolic calcium reuptake in this study. Although these findings indicate a potential mechanism responsible for the functional diastolic impairment recently reported in these same animals (Hiemstra et al. 2013), they do not explain why the level of diastolic dysfunction was greater following CsA treatment given the impact of disease on diastolic calcium handling was equivalent in both the HF and HF‐CsA groups. As a result, we also examined extracellular matrix remodeling as a separate potential mechanism fundamental to the accelerated development of heart failure in HF‐CsA animals. Total fibrosis and collagen expression were increased by the same magnitude in both heart failure groups (Fig. 7), suggesting alterations to extracellular matrix remodeling was not a contributing factor to our findings.

Following these analyses, the question remained – why did CsA treatment accelerate the development of heart failure? The most probable mechanism appears to be a systemic hypertensive response to CsA treatment outlined in our previous report (Hiemstra et al. 2013). Increased systolic, diastolic, and mean arterial pressures observed in the HF‐CsA group likely accelerated the progression to LV dilation in the face of our sustained increase in afterload as a result of chronic aortic‐banding. It is well‐known cyclosporine can cause hypertension (Hoorn et al. 2012), however, the response in our swine HFpEF model significantly exceeded the minimal increase in mean blood pressure (approximately 5 mmHg) previously observed in humans (Robert et al. 2010). Clearly, a hypertensive response of the magnitude observed in this study could drastically increase LV wall tension and subsequent myocardial energetic demand. However, if the hypertensive response to CsA were managed similarly to regular clinical practices for heart failure patients, would the preservation of mitochondrial function observed herein have resulted in a more positive functional outcome for the heart? This is a prudent question regarding the clinical ramifications of our findings and although outside the scope of this study, it is reasonable to speculate that our CsA dosing regimen could improve LV function in a setting of clinically managed hypertension. However, given the numerous detrimental findings found following CsA treatment in our recently published work (Hiemstra et al. 2013) and this study (including the accelerated development of early evidence for heart failure, a significant hypertensive response, a potentially enhanced inflammatory state, and impaired cardiomyocyte calcium handling and contractile function), it is unlikely the use of CsA alone would translate clinically as means to treat HFpEF.

In conclusion, improved mitochondrial function following chronic CsA treatment was not associated with a parallel improvement in cardiomyocyte calcium handling and contractile function in a clinically relevant large animal model of HFpEF. Given CsA accelerated the development of heart failure in our studies at both the whole heart and cellular levels, our findings suggest chronic treatment with CsA alone is unlikely to foster a clinically positive functional outcome in HFpEF patients. Furthermore, our findings demonstrate for the first time in our HFpEF model impaired cardiomyocyte calcium handling and contractile function are present early in the HF disease process. The divergent nature of our findings in comparison to the phase III CIRCUS clinical trial, in which patients are given a one‐time dose of CsA, highlights the importance of dosing strategy (acute vs. chronic) and type of heart failure (HFrEF vs. HFpEF) when considering the use of CsA to clinically manage heart failure.

## Acknowledgments

The authors thank Melissa Cobb, Diana Douglas, Anne Gibson, and Jan Ivey for their considerable technical contributions, which were essential to the successful completion of the study.

## Conflict of Interest

None declared.
